# Assessing heterogeneity in spatial data using the HTA index with applications to spatial transcriptomics and imaging

**DOI:** 10.1093/bioinformatics/btab569

**Published:** 2021-08-06

**Authors:** Alona Levy-Jurgenson, Xavier Tekpli, Zohar Yakhini

**Affiliations:** Department of Computer Science, Technion – Israel Institute of Technology, Haifa 32000, Israel; Department of Medical Genetics, Institute of Clinical Medicine, University of Oslo and Oslo University Hospital, Oslo, Norway; Department of Cancer Genetics, Institute for Cancer Research, Oslo University Hospital, Oslo 0310, Norway; Department of Computer Science, Technion – Israel Institute of Technology, Haifa 32000, Israel; Arazi School of Computer Science, Interdisciplinary Center, Herzliya 4610101, Israel

## Abstract

**Motivation:**

Tumour heterogeneity is being increasingly recognized as an important characteristic of cancer and as a determinant of prognosis and treatment outcome. Emerging spatial transcriptomics data hold the potential to further our understanding of tumour heterogeneity and its implications. However, existing statistical tools are not sufficiently powerful to capture heterogeneity in the complex setting of spatial molecular biology.

**Results:**

We provide a statistical solution, the HeTerogeneity Average index (HTA), specifically designed to handle the multivariate nature of spatial transcriptomics. We prove that HTA has an approximately normal distribution, therefore lending itself to efficient statistical assessment and inference. We first demonstrate that HTA accurately reflects the level of heterogeneity in simulated data. We then use HTA to analyze heterogeneity in two cancer spatial transcriptomics datasets: spatial RNA sequencing by 10x Genomics and spatial transcriptomics inferred from H&E. Finally, we demonstrate that HTA also applies to 3D spatial data using brain MRI. In spatial RNA sequencing, we use a known combination of molecular traits to assert that HTA aligns with the expected outcome for this combination. We also show that HTA captures immune-cell infiltration at multiple resolutions. In digital pathology, we show how HTA can be used in survival analysis and demonstrate that high levels of heterogeneity may be linked to poor survival. In brain MRI, we show that HTA differentiates between normal ageing, Alzheimer’s disease and two tumours. HTA also extends beyond molecular biology and medical imaging, and can be applied to many domains, including GIS.

**Availability and implementation:**

Python package and source code are available at: https://github.com/alonalj/hta.

**Supplementary information:**

Supplementary data are available at *Bioinformatics* online.

## 1 Introduction

This study provides a novel solution for characterizing and statistically assessing spatial heterogeneity. Recently, there has been growing evidence that phenotypical and clonal heterogeneity may play a crucial role in tumour biology and in affecting cancer progression and treatment outcome (AbdulJabbar *et al*., 2020; Ma *et al*., 2020). Cancer cells differ in molecular characteristics such as mutations, gene expression and copy number aberrations. These differences, which define the concept of clonality in tumours, are a potentially detrimental hallmark of cancer. In particular, tumour sub-populations may possess a unique combination of molecular traits that enables them to evade treatment (Dobson *et al*., 2020). The heterogeneous environment arising from such sub-populations has been mainly investigated through bulk measurements. However, bulk measurements lack the spatial dimension, which may harbour potentially critical information. For example, the evolutionary dynamics of cancer may result in tumour subclones residing in distinct microhabitats that support the development of therapy-resistant populations (Gillies *et al*., 2012). In glioblastoma, differences in copy number alterations and somatic mutations were observed when assessing different tumour microenvironments: EGFR-amplified cancer cells were mainly found in poorly vascularized regions, whereas PDGFRA-amplified cancer cells were observed in close proximity to endothelial cells (Little *et al*., 2012). The spatial distribution of immune-cells among tumour cells has a long-standing role in diagnosis (Hendry *et al*., 2017), and was proven useful in predicting prognosis and treatment response in multiple cancer types and molecular settings (Nearchou *et al*., 2020; Zheng *et al*., 2020). Recent advances in spatial transcriptomics, including technology developed for direct measurement [e.g. Visium spatial RNA-sequencing (RNA-seq) by 10x Genomics)], as well as approaches for inferring such information from digital pathology images (Coudray *et al*., 2018; Levy-Jurgenson *et al*., 2020), have accentuated the interest in analyzing molecular heterogeneity from a spatial perspective (Ev Andersson *et al*., 2020; Levy-Jurgenson *et al*., 2020; Masuda *et al*., 2019), with some studies already indicating its potential clinical utility (Levy-Jurgenson *et al*., 2020; Masuda *et al*., 2019).

To support such analyses, we have developed a statistical tool that measures the level of spatial heterogeneity—the HeTerogeneity Average index (HTA). We demonstrate its use using synthetic data, two spatial transcriptomics datasets and four brain MRI scans. We also demonstrate its applicability to other domains.

Several methods have been recently adopted from other fields, mainly ecology, to assist in the quantitative analysis of the spatial heterogeneity of molecular measurements (Yuan, 2016). However, such methods, originating from other fields, do not easily extend to complex biological environments. First, they were mostly designed for univariate and bivariate analyses. For example, Morisita-Horn (Rempala and Seweryn, 2013) is a measure of overlap between two types of elements, such as two species. It has been used in Maley *et al*. (2015) to measure the colocalisation of immune and cancer cells in breast cancer; Moran’s I, and the more recent *q*-statistic (Wang *et al*., 2016), both originating in ecology, measure the spatial auto-correlation and spatial stratified heterogeneity (respectively) of a single attribute with respect to neighbouring locations in space. Another method, Ripley’s K (Ripley, 1976), determines whether a single attribute is dispersed, clustered or randomly distributed in the target spatial environment. Since we are interested in analyzing complex biological environments, with many molecular traits, univariate and bivariate methods fall short of providing an adequate solution (as demonstrated in Section 4). Moreover, these methods may also be difficult to interpret or complex to use (e.g. including edge-correction and radius parameters as in Ripley’s K). Importantly, little is known about the distribution of the null hypothesis for the vast majority of these methods. For Morisita-Horn and Ripley’s K, for example, *P*-values are empirically estimated using Monte-Carlo simulations, which are computationally expensive and less accurate compared to methods based on a known null distribution.

The method we propose in this article, HTA, which is based on Shannon’s entropy, addresses these shortcomings. First, HTA is multivariate, allowing it to capture a richer representation of heterogeneity, even in the bivariate case (see Section 4, Fig. 10); second, it lends itself to easier interpretation since it is based on the notion of entropy; and third, for a fixed set of traits, it requires only a single input parameter. Importantly, the HTA distribution, under a null model, can be well characterized and it thus facilitates efficient statistical assessment and inference.

## 2 Materials and methods

In this section, we introduce HTA—the HeTerogeneity Average Index. We first (Section 2.1) define an index called HTI (HeTerogeneity Index—a variation of Shannon’s entropy) which we will use to measure heterogeneity at a local level. The HTA index (Section 2.2), representing heterogeneity at the whole sample level, will be based on averaging local HTIs. Finally (Section 2.3), we prove that HTA has an approximately normal distribution.

### 2.1 HTI

We first define a heterogeneity index, HTI (Levy-Jurgenson *et al*., 2020), on which HTA is based. HTI is a variation of Shannon’s entropy. Formally:

$$\text{HTI}=-\sum_{c=1}^{C}p_{c}{\;\text{log}\;}_{C}(p_{c})$$

where *C* is the number of non-empty trait combinations that may be observed in the analyzed sample, which typically equals $2^{\left|\text{traits}\right|}-1$ (the number of subsets excluding the empty set); and *p_c_* is the proportion of spatial positions for which exactly all traits in combination *c* manifest. A spatial position, for instance, could be a barcoded spot in spatial transcriptomics data or a tile derived from a pathology whole-slide image as in Coudray *et al*. (2018) and Levy-Jurgenson *et al*. (2020).

For example, for two traits, e.g. FOXA1 and MKI67, whose gene expression levels were spatially resolved to a whole slide image from a breast cancer sample, we have *C *=* *3 for 3 possible non-empty trait combinations: FOXA1 (only), MKI67 (only) and Both. If the tissue is homogeneous with nearly all of its sections falling into one of these three options (say Both), then p(Both)≅1,p(FOXA1)≅0,p(MKI67)≅0 and HTI is 0. If, however the tissue is heterogeneous with 1/3 of the tiles falling into each option then: p(Both)=1/3,p(FOXA1)=1/3,p(MKI67)=1/3 and HTI is 1. The logarithm base *C* guarantees that HTI falls within [0,1]. In this case, a high HTI indicates there may be two or more phenotypically different cell types, whereas a low HTI would reflect single phenotypical dominance.

While HTI was shown to capture heterogeneity at a global level (Levy-Jurgenson *et al*., 2020), it is agnostic to the within-tissue distribution of the trait combinations. For example, the sample in Figure 2 (left), and the sample in Figure 2 (right) have different spatial distributions of the same elements, but HTI is 1 in both cases. This is expected since HTI is a global measure of heterogeneity. However, there is clearly a difference in heterogeneity at the local level, which may have important clinical implications. Our HTA index, described below, which uses HTI at the local level, is designed to capture this difference. Indeed, as noted in Figure 2, the corresponding HTA scores are 0 (homogeneous) on the left and 1 (heterogeneous) on the right.

### 2.2 HTA

#### 2.2.1 HTA definition

**Fig. 1.**
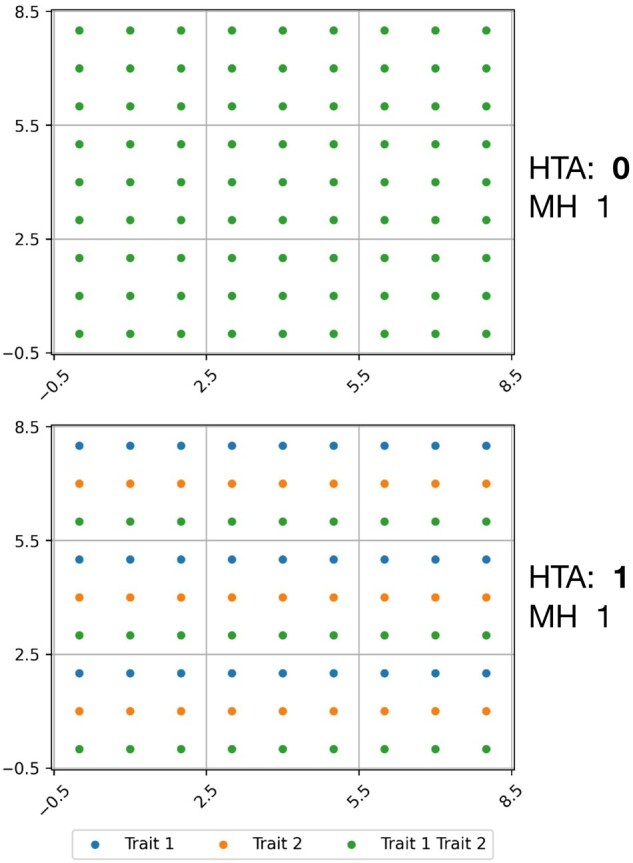
HTA applied to synthetic random data of shape (32, 32) across three different region sizes (2, 8, 16 left-to-right). The data (dot location and color) is held constant across all three. *P*-values demonstrate heterogeneity since *H*_0_ is not rejected (HTA *P*-value > 0.3) for all region sizes

HTA is essentially a weighted average of HTIs across a defined set of spatial regions of a sample (Fig. 1 depicts such regions). To formally describe HTA, we first define what regions of a matrix are, and then use these to define HTA.

**Fig. 2.**
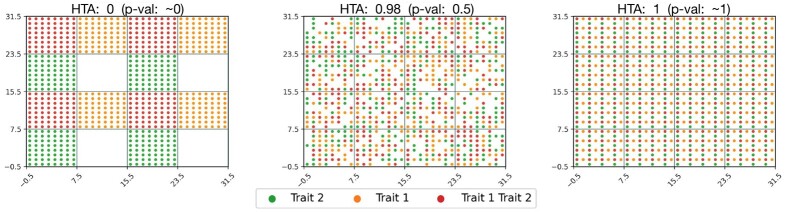
HTA applied to synthetic data across three different distributions (homogeneous, random heterogeneous, deterministic heterogeneous from left-to-right). Region size and trait proportions are held constant (8 and 0.5 resp.). HTA *P*-values: (left) significant homogeneity (p≅0); (middle) not significant (*P *=* *0.23); (right) significant heterogeneity (1-p≅0)

Consider a matrix *M*, where each entry corresponds to a spatial location in the sample [e.g. a barcoded spot from spatial RNA-seq data or a single tile from a pathology image (Levy-Jurgenson *et al*., 2020)] and indicates which of the *C* trait combinations is present therein (or ‘None’ otherwise). Then we consider *M* to be a trait-combination matrix.

#### HTA

Let $M{|}_{G}=\{M_{1},M_{2},\ldots ,M_{R}\}$ be the set of regions obtained by applying grid *G* to a trait-combination matrix *M*. Let $\left\{n_{1},n_{2},\ldots ,n_{R}\right\}$ be the corresponding number of entries in each region that are not ‘None’. Then we define:

(1) $$\mathrm{HTA}(M{|}_{G}):=\sum_{r=1}^{R}\frac{n_{r}}{n}HTI(M_{r})=\sum_{r=1}^{R}\frac{n_{r}}{n}(-\sum_{c=1}^{C}\frac{n_{rc}}{n_{r}}\;\text{log}\;\frac{n_{rc}}{n_{r}})$$

where *n_rc_* is the number of entries in region *r* that manifest trait combination c∈{1,…,C}, and *n* is the total number of entries, in the entire matrix, that manifest at least one trait.

Note that since *n_r_* is the number of entries in region *r* that are not ‘None’, this means that $\sum_{c=1}^{C}\frac{n_{rc}}{n_{r}}=1$, for all *r*. Empty regions (where all elements in the region are ‘None’) are discarded.

**Fig. 3.**
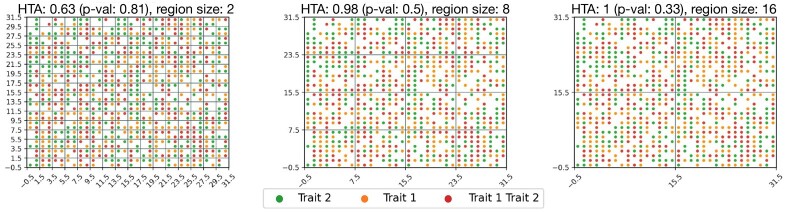
Heterogeneity maps and corresponding HTAs for: (**B–D**) two traits: ESR1 and GATA3; (**F–H**) three traits: ESR1, GATA3 and FOXA1. Each color represents the manifestation of a different trait combination. In B–D, red means that ESR1 and GATA3 are highly expressed (above their respective medians), green—only GATA3 is highly expressed and orange—only ESR1 is highly expressed. In F–H, due to the large number of trait-combinations, we note here the most common trait combinations and provide the full legend in Supplementary Material S4: grey—all three traits are highly expressed, pink—GATA3 and FOXA1 are highly expressed, red—ESR1 and GATA3 are highly expressed. HTA is significantly homogeneous at all region sizes (5, 15 and 30) in both settings (HTA *P*-values $<10^{-8}$). This aligns with the expected outcome for this cancer type (Luminal B breast cancer, for which the cancer cells highly express these three transcription factors); (**A**) and (**E**): the resulting heterogeneity maps if the respective trait-combinations were randomly distributed (*H*_0_). HTA *P*-values are 0.26 and 0.56 (A, E respectively) at region size 5, as expected under *H*0

For example, in spatial RNA-seq, each entry in *M* that is included in *n* represents a barcoded spot that manifests at least one trait (e.g. the coloured dots in Fig. 3D), and each included region of *M* contains several such barcoded spots (e.g. the non-empty regions bordered by the grid lines in the same figure). In digital pathology, where each whole-slide image is divided into thousands of smaller images (tiles), each entry represents a single tile in which at least one trait is present and each region of *M* contains several such tiles.

**Fig. 4.**
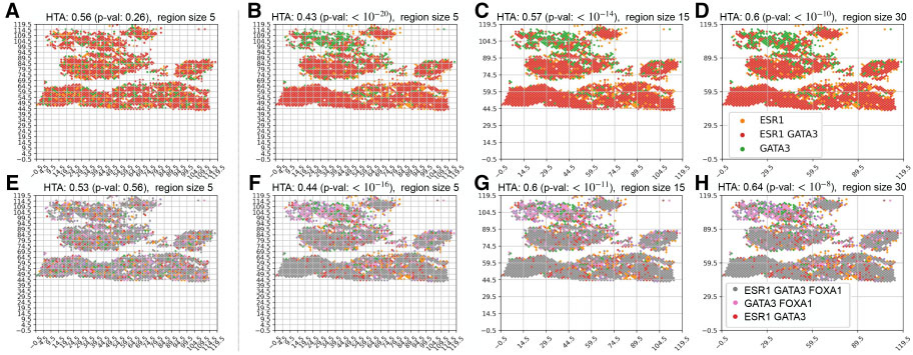
(**B–D**) Heterogeneity maps and HTA depicting the mutual distribution of ERBB2 (HER2) and CD8A (T-cells) for the bottom half of Figure 3, chosen due to indications of high tumour content in that portion (see Section 3.2). (**A**) The resulting heterogeneity map if ERBB2 and CD8A were randomly distributed (*H*_0_). HTA is: (B–C) significantly homogeneous $p<10^{-6}$; (D) heterogeneous *P *=* *0.1 (*H*_0_ not rejected); (A) heterogeneous *P *=* *0.47

We note that HTA monotonically decreases with grid refinement. This is similar to the fact that:

(2) H(Y|X)≤H(Y)

for any random variables *X* and *Y* (for a proof see Supplementary Material S1). Indeed, we observe this in Figure 1, where HTA decreases from right-to-left. We note that since region-based methods are inherently sensitive to region size, HTA’s monotonicity provides an added advantage since it guarantees an ordering one can expect to observe when moving between region sizes. In Figure 4, for example, we can see that for the largest region size (D), the null hypothesis of heterogeneity is not rejected (HTA *P *=* *0.1), whereas at the smaller region sizes (B–C) it is. Knowing that HTA monotonically decreases with grid refinement, a user may be inclined to test finer grids before concluding that the sample is heterogeneous with respect to the mutual spatial distribution of T-cells among HER2 cells.

### 2.3 HTA *P*-value

We compute the HTA *P*-value under the null model in which all trait combinations are uniformly distributed across the tissue sample, as in Figures 3A, E and 4A (i.e. a random permutation of the exact trait combinations present within the tissue sample, naturally preserving the observed frequencies).

We use a permutation over trait-combinations, rather than a permutation over individual traits, since only the former preserves the natural trait-combinations present within the sample. To illustrate the difference, consider a sample that exhibits only one type of cell, say one that over-expresses all of: KRAS, BRAF, EGFR and TP53. Under a trait-based permutation, this single trait-combination could turn into $2^{4}-1$ trait-combinations (all non-empty subsets), changing the true underlying molecular composition. Conversely, under a permutation over trait-combinations, only the original trait-combination would be present, alleviating this problem.

Note that since the null model depends on the original trait-combination composition, lower HTA values do not necessarily correspond to lower HTA *P*-values (see Supplementary Material S7). As such, the interpretation of results should rely on the *P*-value rather than on the statistic, as in many standard statistical tests.

#### 2.3.1 Equal-weight regions

If we assume that all regions contain the same number of entries, we obtain that HTA is normally distributed, by the classical central-limit theorem (Lindeberg–Lévy CLT). Formally, we denote *X_r_*, r=1,…,R to be $\mathrm{HTI}(M_{r})$. Then, under the null hypothesis, the random variables *X_r_* are iid. Therefore, by the CLT, their mean (HTA) is normally distributed:

(3) $$\frac{1}{\sigma /\sqrt{R}}\left({\bar{X}}_{r}-\mu \right)\overset{d}{\underset{}{\to }}N(0,1)$$

where *μ* and *σ* are the mean and standard-deviation of *X_r_*, under the null model.

In our case this means:

(4) $$\frac{1}{\sigma /\sqrt{R}}\left(\frac{1}{R}\sum_{r=1}^{R}X_{r}-\mu \right)=\frac{1}{\sigma /\sqrt{R}}\left(HTA-\mu \right)\overset{d}{\underset{}{\to }}N(0,1)$$

*μ* and *σ* depend on both the region size and the distribution of trait combinations in the matrix *M*. We can estimate these quantities from simulations of *M* under the null model. For a limited region size, we can also compute these quantities precisely by running an exhaustive search across the permutations of trait combinations in a single region to obtain all possible values for *X_r_* and its resulting distribution.

Using this approach for two traits (3 non-empty trait combinations, *C *=* *3) and region sizes 2 and 3 (yielding square regions with $2^{2}=4$ and $3^{3}=9$ elements, respectively) we obtain, for example, that for sufficiently large values of *R* the following holds (respectively):

$$\begin{array}{c} HTA∼N(0.57,\frac{0.31^{2}}{R})\;(\mathrm{region}\;\mathrm{size}=2)\\ HTA∼N(0.83,\frac{0.17^{2}}{R})\;(\mathrm{region}\;\mathrm{size}=3) \end{array}$$

#### 2.3.2 Weighted regions

For actual data, the number of entries in each region may vary as a function of the positions in which entries of *M* are empty. Their corresponding HTIs are therefore no longer identically distributed under the null hypothesis. Specifically, we have different means and stds for the HTI of each of the regions, indexed by *r*, which we denote by *μ_r_* and *σ_r_*, respectively. For example, a region with only one entry will always exhibit a single trait combination, leading to HTI =0 and therefore $\mu_{r}=0$, whereas a region with more entries has a positive probability of obtaining a non-zero HTI and therefore $\mu_{r}>0$. Since the classical CLT (Lindeberg–Lévy CLT) assumes that the random variables are iid, we turn to a different version of CLT that applies to independent, but not identically distributed, random variables—the Lyapunov CLT:

#### Lyapunov CLT

Let $X_{1},X_{2},\ldots X_{m}$ be independent random variables with $EX_{i}=\mu_{i}$ and $\text{Var}X_{i}=\sigma_{i}^{2}<\infty$. Denote $Y_{i}=X_{i}-\mu_{i}$.

(Lyapunov Condition) If there exists δ>0 such that:

(5) $$\underset{m\to \infty }{\lim}\frac{1}{s_{m}^{2+\delta }}\sum_{i=1}^{m}E(|Y_{i}{|}^{2+\delta })=0$$

where

(6) $$s_{m}^{2}=\text{Var}(\sum_{i=1}^{m}Y_{i})=\sum_{i=1}^{m}\sigma_{i}^{2}$$

then

(7) $$\frac{1}{s_{m}}\sum_{i=1}^{m}\left(X_{i}-\mu_{i})\overset{d}{\underset{}{\to }}N(0,1\right)$$

In our case, we want to use this theorem with $X_{i}=\frac{n_{r}}{n}HTI(M_{r})$ and *m *=* R* and obtain:

(8) $$\frac{1}{s_{R}}\left(HTA-\sum_{r=1}^{R}\mu_{r}\right)\overset{d}{\underset{}{\to }}N(0,1)$$

We observe that the Lyapunov condition is satisfied in our case. For any δ>0,

(9) $$E|Y_{r}{|}^{2+\delta }\leq EY_{r}^{2}\leq 1$$

because $Y_{r}\in [0,1]$ (since HTI ∈[0,1]).

Therefore

(10) $$\frac{1}{s_{R}^{2+\delta }}\sum_{r=1}^{R}E|Y_{r}{|}^{2+\delta }\leq \frac{1}{s_{R}^{2+\delta }}\sum_{r=1}^{R}\text{Var}Y_{r}=\frac{1}{s_{R}^{\delta }}$$

where the first inequality follows from Equation 9 combined with the fact that $\text{Var}Y_{r}=EY_{r}^{2}$ and the equality follows immediately from the definition of $s_{R}^{2}$ (Equation 6).

It remains to show that $s_{R}\to \infty$ as R→∞. Indeed, under the null hypothesis, the set of variances ${\left\{\sigma_{r}^{2}\right\}}_{r=1}^{R}$ is bounded away from zero if we assume that there are no single-sample regions (otherwise we may increase the region size), or that there is a constant number of such regions.

Given a specific dataset, in order to use Lyapunov CLT, we must estimate *μ_r_* and *σ_r_* for all relevant (non-empty) regions, r=1,…,R. We do so by simulating 1000 random-uniform permutations of the trait combination matrix (while holding constant the original positions of non-empty elements) and for each permutation we compute HTIs for all relevant regions. Then, for each region r∈{1,…,R}, we use its 1000 HTIs to estimate *μ_r_* and *σ_r_*.

We emphasize that the normal approximation holds only for sufficiently large values of *R*. We also note that for adequate interpretation of the HTA results, one should consider two one-sided *P*-values. Namely *P* and 1−P, which represent the alternative hypotheses of homogeneity and heterogeneity, respectively. To determine the overall significance, the smaller *P*-value should be doubled.

## 3 Results

In this section, we demonstrate the use of HTA in several domains. We begin with synthetic data for both 2-dimensional and 3-dimensional spatial data (Section 3.1). We then apply HTA to two 2-dimensional spatial transcriptomics datasets: Visium spatial RNA-seq by 10x Genomics (Section 3.2) and spatial transcriptomics inferred from pathology whole-slide images (Section 3.3). We also demonstrate a 3-dimensional use case using MRI images (Section 3.4). Finally, we demonstrate that HTA extends to other domains by analyzing US census data (Section 3.5).

### 3.1 Synthetic data

In Figures 1 and 2, we depict results from applying HTA to 2-dimensional heterogeneity maps of shape (32, 32), each of which represents a trait-combination matrix. Regions are the visible square areas that fall between the grid lines. In Figure 1, we see heterogeneity maps for two traits, each of which has a random uniform distribution with probability 0.5 of occurrence in each position. As one would expect, the null hypothesis that the trait-combinations are randomly distributed is not rejected in all three region sizes (2, 8 and 16) since HTA *P*-values are >0.3.

Figure 2 demonstrates that HTA discerns between homogeneous and heterogeneous distributions. Holding the region size constant, we observe that a perfectly homogeneous distribution within each region (left) is significantly homogeneous (HTA *P*-value ≅0), while a perfect heterogeneous distribution (right) is significantly heterogeneous (HTA 1-*P*-value ≅0). In comparison, a random heterogeneous distribution from *H*_0_ (middle) is neither (HTA *P*-value 0.5). This means that both significant homogeneity and significant heterogeneity are identified using HTA.

**Fig. 5.**
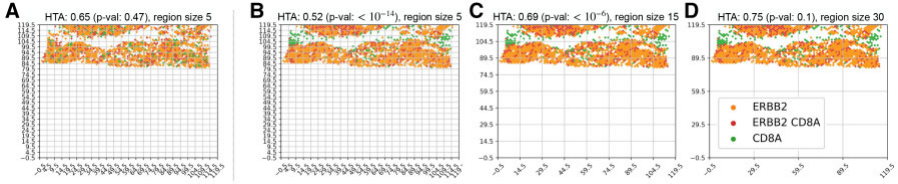
A 3D heterogeneity map and HTA for two region sizes that differ along the *z*-axis. The two illustrations at the bottom illustrate the resulting regions along with the corresponding HTA results: (left) region size (8, 8, 1) each region is perfectly homogeneous, yielding HTA 0 and HTA *P*-value ≅0; (right) region size (8, 8, 3)—each region is perfectly heterogeneous, yielding HTA 1 and HTA *P*-value ≅1. Note that the 3D grid visible at the top does not describe the regions

In Figure 5 we applied HTA to a 3-dimensional heterogeneity map of shape (32, 32, 3) (for the *x*, *y*, *z* axes, respectively). Since the region size can now also vary across the *z*-axis, we use two region sizes that differ only along this axis: (8, 8, 1) and (8, 8, 3) as illustrated at the bottom of Figure 5. Using a region size of (8, 8, 1), as in Figure 5 (left), where each region manifests exactly one of the trait combinations, we obtain significant homogeneity (HTA *P*-value ≅0). Conversely, using a region size of (8, 8, 3), illustrated in Figure 5 (right), where each region contains an equal amount of each of the three trait combinations, we obtain significant heterogeneity (HTA *P*-value of ≅1).

### 3.2 Spatial RNA sequencing

We use 10x Genomics’ Visium breast cancer spatial gene-expression data (see Supplementary Material S2 for details). The sample is a Stage Group IIA breast cancer of type Luminal B (ER positive, PR negative and HER2 positive). To determine *n_rc_*, the number of entries in region *r* manifesting trait-combination *c*, we use the median threshold for each gene. An entry manifests combination *c* if all the genes in this combination are above their respective median expression level. Since this sample is Luminal B, it is expected that ESR1, FOXA1 and GATA3 would be spatially co-expressed (Jacquemier *et al*., 2009), leading to a significantly homogeneous HTA index. We tested both two and three traits. Indeed, in Figure 3B–D, we observe that the tissue sample is significantly spatially homogeneous at both a local and global resolution (smaller and larger region-sizes, respectively), obtaining HTA *P*-values of $<10^{-10}$. This is compared to a random permutation of the observed trait combinations under *H*_0_ (Fig. 3A) which obtains an HTA *P*-value of 0.26 even at the smallest region size of 5. For the three traits: ESR1, GATA3 and FOXA1 (Fig. 3F–H) we observe similar results, with HTA *P*-value of $<10^{-8}$ at all three region sizes. This is in comparison to the random permutation of the observed trait combinations (Fig. 3E) which obtains an HTA *P*-value of 0.56 at region size 5.

Previous research has shown that T-cells remaining at the periphery of cancer cells, with low tumour infiltration, may be indicative of poor prognosis compared to tumours with high T-cell infiltration (Pruneri *et al*., 2018). HTA can help identify such cases. In Figure 4B–D we generated the heterogeneity map for ERBB2 (HER2) and CD8A (T-cells) using the same HER2 positive breast cancer sample. We focused on the area where ESR1 and GATA3 are relatively co-expressed (bottom half of the tissue in Fig. 3) since, as explained above, these regions are likely to have high tumour content. Using HTA, we observe significant homogeneity in the two smaller region-sizes, 5 and 15, with HTA *P*-values $<10^{-6}$. Interestingly, at the larger region size of 30 we no longer observe significant homogeneity, with a *P*-value of 0.1. For context, a random dispersion of these T-cells (Fig. 4A) is not significantly homogeneous, with an HTA *P*-value of 0.47. The significant homogeneity at the two smaller region sizes mean that the tumour cells are rarely, if at all, infiltrated by T-cells. However, the lack of significant homogeneity at the larger region size indicates that there is at least some infiltration of T-cells (otherwise we would observe significant homogeneity at this level too).

**Fig. 6.**
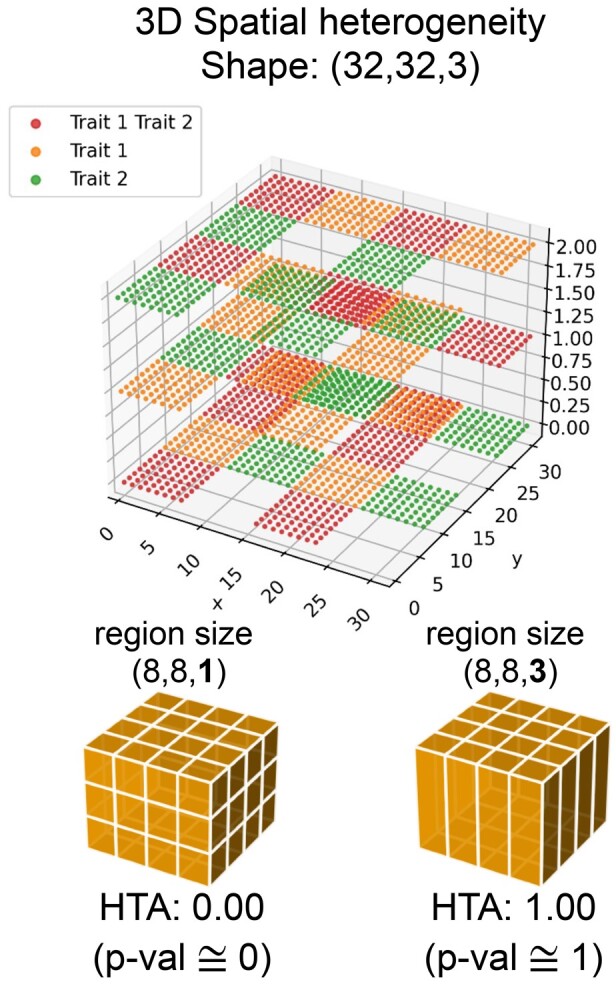
Heterogeneity maps and HTA for seven breast cancer driver genes: MYC, ESR1, ERBB2, GATA3, FOXA1, TP53 and CDK4. (**A**) Actual heterogeneity map, with HTA *P*-value of $10^{-16}$ at region size 15; (**B**) heterogeneity map under *H*_0_ (random permutation of the trait-combinations), with HTA *P*-value of 0.43

Since HTA is designed to handle a large number of traits, it is capable of capturing certain characteristics of clonal composition. We demonstrate this using the same breast cancer sample and 7 breast cancer driver genes: MYC, ESR1, ERBB2, GATA3, FOXA1, TP53 and CDK4. In Figure 6A we can see the resulting heterogeneity map. Using a region size of 15, we obtain a significantly homogeneous HTA (*P*-value: $10^{-16}$). In B we observe a random permutation of the observed trait combinations, which results in a non-significantly homogeneous HTA (*P*-value: 0.43). Since 7 traits give rise to $2^{7}-1=127$ non-empty combinations (provided that all are present), we do not attempt to display the legend. Instead, we produce a ‘region-report’ to identify the most frequent combinations in regions of interest. For example, in the bottom left corner, at (−0.5,59.5), the most frequent combination is all 7 driver genes, accounting for 73% of the elements in that region. The second most frequent combination is the 6 driver genes that remain after removing CDK4. While the region to its right has a similar composition, two regions to the right, at (29.5,59.5), already exhibits a different, yet relatively homogeneous composition: the combination of all 7 account for 41% of the elements; a small number of different combinations of 6 of the driver genes account for 26% and; the vast majority of the remaining elements (accounting for 28%) are several combinations of 4 and 5 genes. These observations align with the significantly homogeneous HTA, and may indicate that the significant homogeneity may be due to the gradual formation of a dominant subclone that over-expresses all 7 genes.

### 3.3 Spatial transcriptomics from pathology whole-slide images

**Fig. 7.**
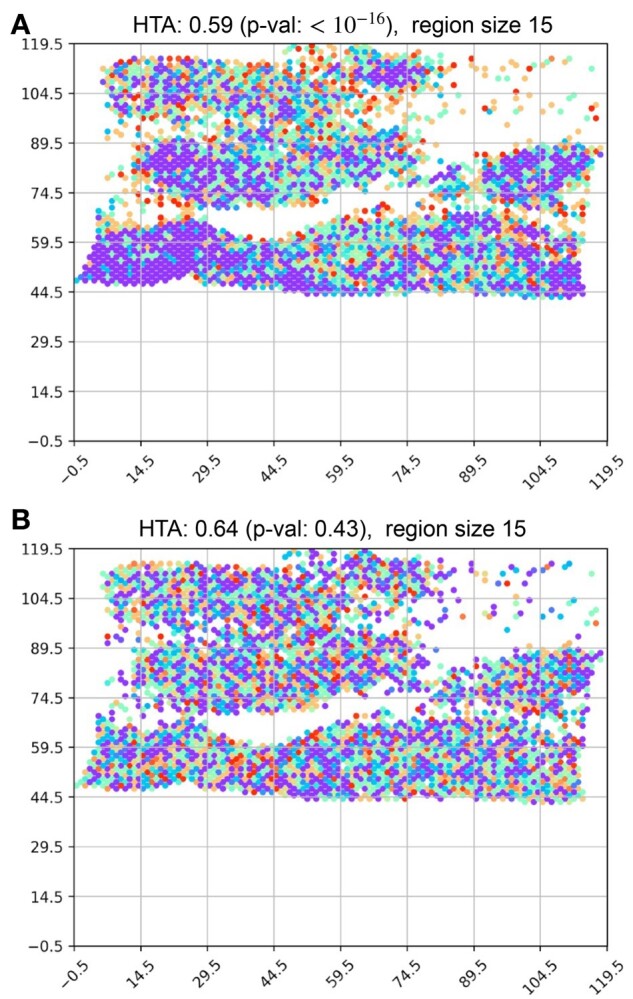
Survival analysis with respect to HTA derived from two spatially resolved traits in breast cancer pathology whole-slides—MKI67 and miR-17 expression level. Binary spatial transcriptomics maps, inferred from the slides of 324 subjects, were split into high and low HTA with respect to the cohort’s median: > median (blue) and ≤ median (orange). The plots differ in the region size used to compute HTA. HTA region sizes and corresponding log-rank *P*-values: (**A**) 15, *P *=* *0.15; (**B**) 30, *P *=* *0.01; (**C**) 45, *P *=* *0.06. All maps were resized to (90, 90), close to the median map size, using nearest neighbour interpolation. Each survival curve is shown with a 95% confidence interval

In this section, we use spatial transcriptomics inferred from breast cancer pathology whole-slide images obtained from Levy-Jurgenson *et al*. (2020). This dataset contains 324 subjects for which: (i) MKI67 and miR-17 expression were spatially resolved to their respective pathology whole-slide images, yielding binary maps that indicate where each gene was detected as over-expressed; and (ii) survival data is available. Since not all slides, and therefore inferred maps, have the same size, we first resize them to (90, 90), chosen based on their median shape. To ensure that the maps remain binary, we use nearest neighbour interpolation during resizing. We apply HTA using the three region sizes: 15, 30 and 45. We then split the cohort into two equal sets based on their HTAs: > median HTA and ≤ median HTA (relatively heterogeneous and relatively homogeneous, respectively). Figure 7 shows the results for the survival analysis performed using these heterogeneity-based assignments. We observe significant survival differences in the two larger region sizes, with the middle one, region size 30, obtaining the lowest *P*-value of 0.01.

### 3.4 MRI

**Fig. 8.**
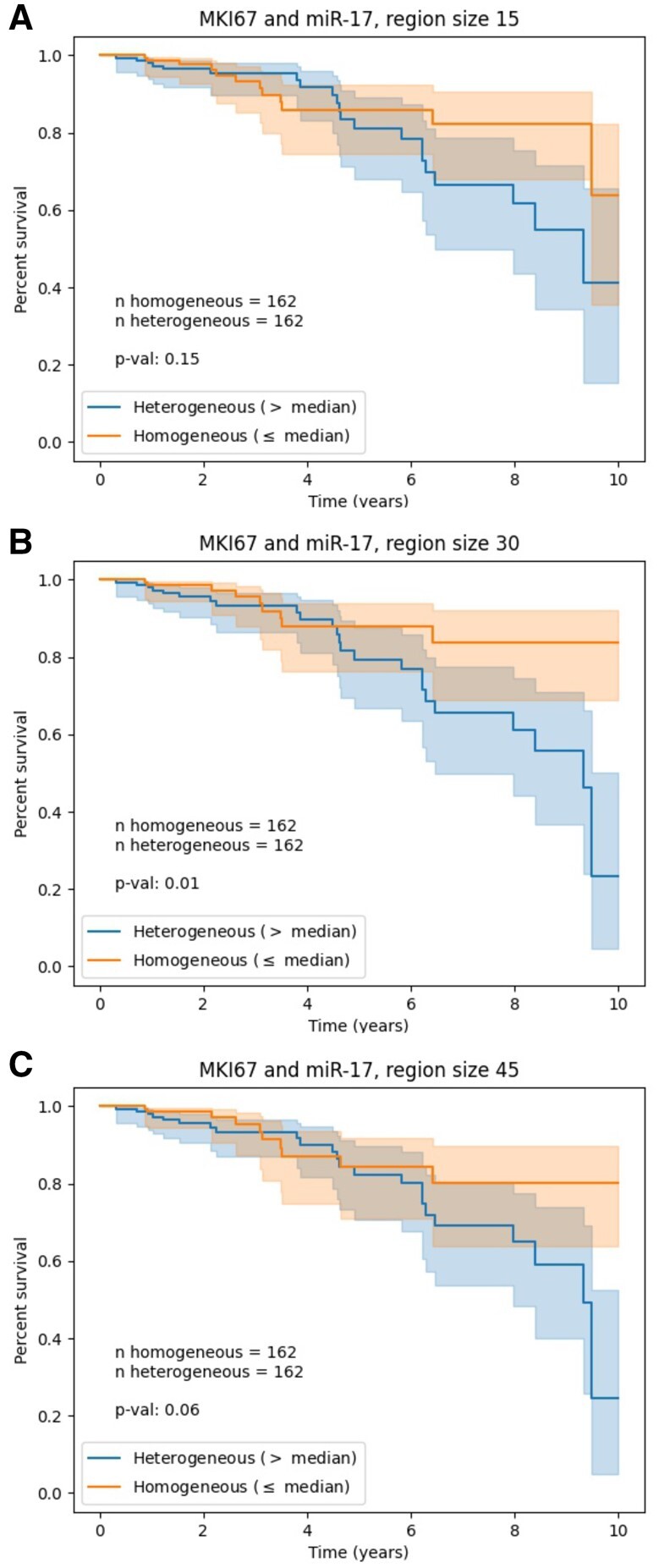
Heterogeneity maps and HTA for four MRI scans taken from different subjects. The shape of each heterogeneity map is (256, 256, 3) and the region size is (128, 128, 3). From top to bottom (highest to lowest HTA *P*-values): (i) normal ageing, (ii) Alzheimer’s disease, (iii) Metastatic bronchogenic carcinoma and (iv) Glioma

In this section, we demonstrate the use of HTA in the context of 3D data by analyzing brain MRI scans (see Supplementary Material S3 for further details). We use four axial MRI scans from four different subjects, representing: (i) normal ageing, (ii) Alzheimer’s disease (iii) Metastatic bronchogenic carcinoma and (iv) Glioma. For each subject, we obtained the only two weighted sequences that were available for all four: T2-weighted and Proton density (PD) weighted (these accentuate different properties; for details see, e.g. Chong *et al*., 2016). We use these as the traits, where a strong signal (brighter) gets 1 and a low signal (darker) gets 0, depending on whether they are above or below the median grayscale value, correspondingly. Since MRI scans are sequences of images (slices), they represent a 3-dimensional space. We use three different slices per subject taken from similar locations in each (Supplementary Material S3). We use three for visualization purposes, but the analysis applies to any number of slices. In Figure 8 we see the resulting 3D heterogeneity maps and HTA for all four subjects. The shape of each map is (256, 256, 3) and the region size is (128, 128, 3). Normal ageing (top) is the only one that is not significantly homogeneous at a 0.01 threshold (*P*-value 0.02). The remaining three, each of which represents a different disease, are significantly homogeneous. The figures are ordered in decreasing *P*-values. Interestingly, the two cancer scans obtain the strongest significance, with *P*-values $10^{-84}$ (metastatic bronchogenic carcinoma) and $10^{-149}$ (glioma). Alzheimer’s disease is also significantly homogeneous, with a *P*-value of 0.0002.

We note that for images, there may be other binarization techniques besides the median that are worth considering (e.g. Li thresholding to detect background versus foreground). Specifically for MRI, it may also be relevant to use actual raw signal measurements, provided such data is available. In such a case, other binarization methods may be better suited than the median value threshold. We have kept the median threshold for simplicity of demonstration.

### 3.5 Census data

**Fig. 9.**
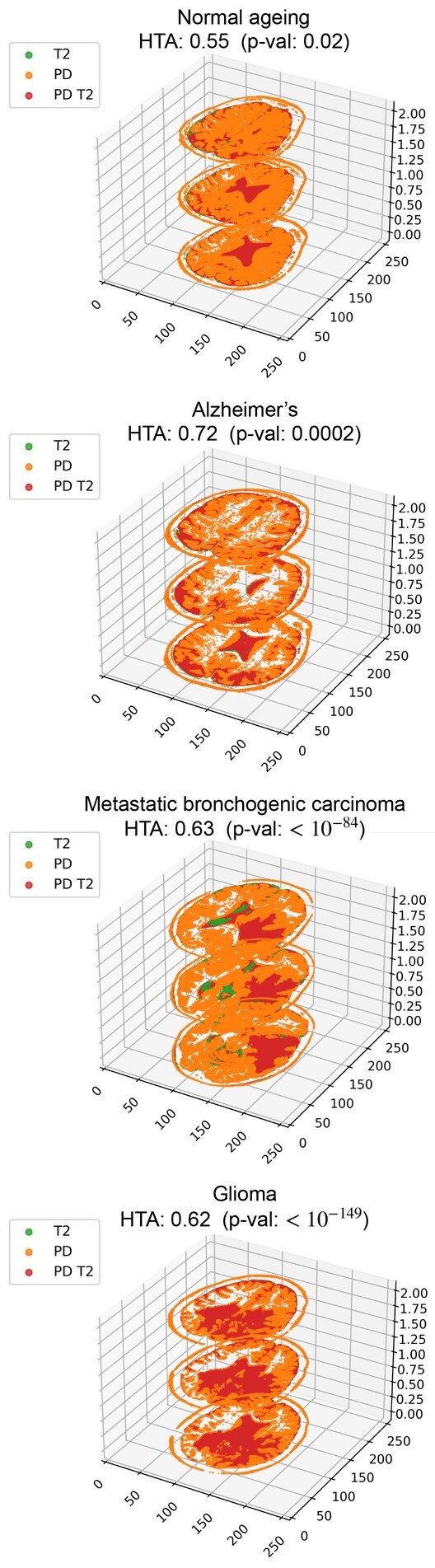
Heterogeneity maps and HTA for US census data for three ethnicity-related traits: ‘Black or African American alone > 5% of total population in county’, ‘Asian alone > 5% of total population in county’ and ‘American Indian and Alaska Native alone > 5% of total population in county’ using region size 70. We observe significant homogeneity with HTA *P*-value $<10^{-19}$

To demonstrate the overall utility of HTA we also applied it to recent census data for 3092 counties across the US, excluding those in Alaska, Hawaii and Puerto Rico due to their relatively large distance from the other states (see Supplementary Material S5). For each county, the data contains the total population per ethnicity, across multiple ethnicity groups. In Figure 9 we observe the heterogeneity map and corresponding HTA index and *P*-value obtained for the three ethnicity-related traits: ‘Black or African American alone >5% of total population in county’, ‘Asian alone > 5% of total population in county’ and ‘American Indian and Alaska Native alone >5% of total population in county’ for region size 70. We observe significant homogeneity, with HTA *P*-value $10^{-19}$, indicating that people from the same ethnic origin tend to cluster in specific regions. Since all regions included over 5% ‘White alone’ we did not include this category.

Since the size of such maps could potentially be much larger, we also tested whether 100 random-uniform permutations (required to compute Lyapunov CLT parameters, as described in Section 2.3.2), instead of 1000, would be sufficient to obtain similar HTAs and *P*-values. The results for 100 repeats are nearly identical to those of 1000 repeats: we obtain *P*-values $10^{-19}$ for 1000 and $10^{-18}$ for 100.

## 4 Discussion

**Fig. 10.**
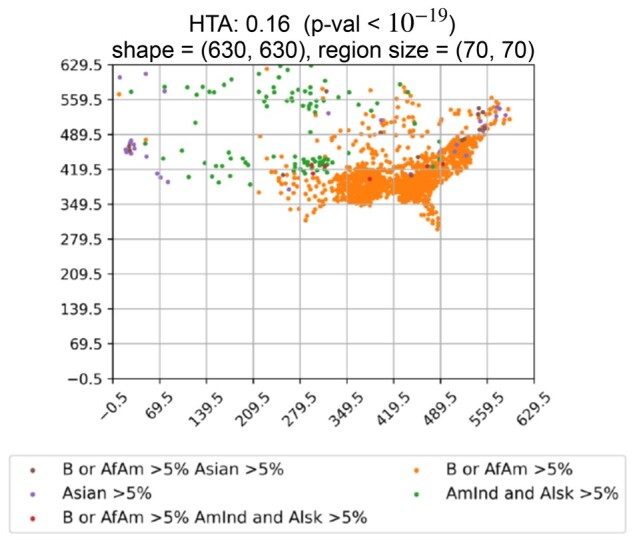
HTA captures a richer representation of heterogeneity, allowing it to differentiate between types of overlap (a proxy used for heterogeneity), even in the bivariate case

HTA provides a solution to the growing need for statistical analysis tools that are capable of quantifying spatial heterogeneity in the complex setting of high throughput molecular biology data, including spatial transcriptomics and digital pathology. It is also useful in other domains, including imaging and geographic information systems. HTA accurately reflects spatial heterogeneity at multiple resolutions, can handle a large number of variables (trait-combinations) and lends itself to efficient statistical assessment. In the context of addressing multiple traits, we also note the difference between HTA and the existing literature. For example, Figure 10 demonstrates that Morisita-Horn, which measures the overlap between two traits across all regions of a given space, will declare a perfect overlap for both examples shown in the figure, whereas HTA successfully discerns between the two.

While HTA is multivariate, it may not easily scale to hundreds or thousands of molecular traits. This can be overcome by using aggregation methods to obtain several meta-traits, each representing a group of individual traits. We demonstrate this in Supplementary Material S6 using immune pathway enrichment scores [computed using GSVA (Hänzelmann *et al*., 2013)], representing a total of 69 genes, and using cluster IDs obtained from 10X’s Loupe Browser, representing 2786 genes. Using such aggregation methods HTA can be applied to data that spans a large number of individual traits.

We have also shown that HTA can be used at multiple scales, controlled by the region-size parameter. We note that other multi-scale measures, such as 2D multi-scale entropy (e.g. Silva *et al*., 2018), as well as fractal-based and wavelet-based methods (e.g. Watanabe *et al*., 2021), while multi-scale, do not apply to the trait-combination representation that HTA can analyze. These methods apply either to 2D images, with a strong emphasis on relationships between colours, or to 2D binary matrices. Since the colours in the heterogeneity maps are not ordinal, and since there is more than one trait-combination, neither option is relevant.

HTA also has other advantages. First, HTA is simple to use since it requires a single input parameter (region size) and easy to interpret since it is directly derived from Shannon’s entropy. This is in contrast to available tools that are limited in at least one of these aspects. HTA also applies to any d-dimensional space. We demonstrated this in 3-dimensions using both synthetic and MRI data. Finally, HTA extends beyond the scope of molecular biology and medical imaging and can be used in many other domains, as was demonstrated with US census data.

HTA can be further used in down-stream analyses. For example, in the case of ERBB2 (HER2) and CD8A (T-cells) demonstrated in Figure 4, different HTAs could potentially be associated with different responses to immune therapy treatment. Results of applying HTA in digital pathology show that HTA may be predictive of survival in breast cancer.

HTA offers many potential extensions worthy of investigation in future work. One option is to combine HTA scores from multiple scales into a new measure that summarizes them. It may also be relevant to extend HTA to apply to continuous measurements. Finally, alternative permutation-based null models may also be investigated. One option is to further investigate the utility of a trait-based permutation (see Section 2.3). Another option is a locality preserving null model (where the region can be determined by some radius), which may be useful in certain cases where retaining local characteristics is important. We note, however, that it may not be appropriate when considering more global phenomenons. One such case is when measuring the level of T-cell infiltration, as demonstrated in Figure 4; had we performed a locality preserving permutation, we would not have been able to assess the significance of infiltration since such a permutation would cause the null model to assume that there *is* T-cell infiltration to begin with (this sample has immune cells around cancer cells in most locations). Nevertheless, alternative null models may be highly relevant in other cases, and offer interesting opportunities for further investigation.

As spatial transcriptomics data and digital pathology inference techniques become increasingly available and accurate, we expect methods that address spatial distributions, including HTA and its potential extensions, to become ubiquitous.

## Supplementary Material

btab569_Supplementary_Data
