# Erratum to: Assessing heterogeneity in spatial data using the HTA index with applications to spatial transcriptomics and imaging

**DOI:** 10.1093/bioinformatics/btab692

**Published:** 2021-10-27

**Authors:** Alona Levy-Jurgenson, Xavier Tekpli, Zohar Yakhini

*Bioinformatics* (2021), https://doi.org/10.1093/bioinformatics/btab569

Upon the original publication of this manuscript, errors were noted. This erratum has been published to address the following changes introduced due to production error and that have subsequently been corrected:

All URL links for the figures have been corrected to link to the appropriated figures.

Under sub-section “2.3.1 Equal-weight regions”, the below equation has been updated as follows:

Previous version:

$$\begin{array}{c} HTA∼N(0.57,\;\frac{0.31^{2}}{R})\;\;(\mathrm{region}\mathrm{size}=2)\\ HTA∼N(0.83,\;\frac{0.17^{2}}{R})\;\;(\mathrm{region}\mathrm{size}=3) \end{array}$$

Corrected version:

$$\begin{array}{c} HTA∼N(0.57,\;\frac{0.31^{2}}{R})\;\;(\mathrm{region}\;\mathrm{size}=2)\\ HTA∼N(0.83,\;\frac{0.17^{2}}{R})\;\;(\mathrm{region}\;\mathrm{size}=3) \end{array}$$

The Publisher would like to apologize for these errors.

